# Fatty acid-binding protein 4 impairs the insulin-dependent nitric oxide pathway in vascular endothelial cells

**DOI:** 10.1186/1475-2840-11-72

**Published:** 2012-06-18

**Authors:** Gemma Aragonès, Paula Saavedra, Mercedes Heras, Anna Cabré, Josefa Girona, Lluís Masana

**Affiliations:** 1 Research Unit on Lipids and Atherosclerosis, Universitat Rovira i Virgili, IISPV, Spanish Biomedical Research Centre in Diabetes and Associated Metabolic Disorders (CIBERDEM), Reus, Spain; 2 Vascular Medicine and Metabolism Unit, Research Unit on Lipids and Atherosclerosis, Sant Joan University Hospital, Universitat Rovira i Virgili, C. Sant Llorenç 21, Reus, 43201, Spain

**Keywords:** Diabetes, Endothelium, Fatty acid-binding protein 4 (FABP4), Endothelial dysfunction, Insulin, Insulin-signalling pathway, Endothelial nitric oxide synthase (eNOS), Nitric oxide (NO)

## Abstract

**Background:**

Recent studies have shown that fatty acid-binding protein 4 (FABP4) plasma levels are associated with impaired endothelial function in type 2 diabetes (T2D). In this work, we analysed the effect of FABP4 on the insulin-mediated nitric oxide (NO) production by endothelial cells *in vitro.*

**Methods:**

In human umbilical vascular endothelial cells (HUVECs), we measured the effects of FABP4 on the insulin-mediated endothelial nitric oxide synthase (eNOS) expression and activation and on NO production. We also explored the impact of exogenous FABP4 on the insulin-signalling pathway (insulin receptor substrate 1 (IRS1) and Akt).

**Results:**

We found that eNOS expression and activation and NO production are significantly inhibited by exogenous FABP4 in HUVECs. FABP4 induced an alteration of the insulin-mediated eNOS pathway by inhibiting IRS1 and Akt activation. These results suggest that FABP4 induces endothelial dysfunction by inhibiting the activation of the insulin-signalling pathway resulting in decreased eNOS activation and NO production.

**Conclusion:**

These findings provide a mechanistic linkage between FABP4 and impaired endothelial function in diabetes, which leads to an increased cardiovascular risk.

## Introduction

The adipose fatty acid-binding protein (FABP), also known as FABP4 and aP2, is one of the best characterised intracellular lipid transport proteins [1]. It belongs to the superfamily of low-molecular-weight intracellular lipid-binding proteins and plays a central regulatory role in energy metabolism and inflammation [2,3]. FABP4 is highly expressed in mature adipocytes and accounts for approximately 6 % of their soluble protein. FABP4 is also found circulating in the plasma. In the last several years, much effort has been focused on uncovering the role of FABP4. However, neither the secretory pathways nor the functions of circulating FABP4 are known. We and other authors have shown that FABP4 levels are increased in obesity, metabolic syndrome (MS), type 2 diabetes (T2D), and familial combined hyperlipidaemia or lipodystrophy syndromes, and these levels are also closely correlated with adverse lipid profiles and insulin resistance [4-10]. In these studies, serum FABP4 predicted the development of MS and atherosclerosis [11,12].

A recent study showed that although serum levels of both adipocyte and epidermal FABP had associations with MS, only FABP4 was significantly associated with increased cardiovascular risk in Chinese adults [13]. Moreover, increased plasma levels of FABP4 in non-elderly men were independently associated with the presence of coronary artery disease [14]. A recent study showed that FABP4 had a direct impact on decreasing the contractility of myocardial muscle cells, which suggested that the release of FABP4 in to the bloodstream could have a direct effect on some peripheral cells and tissues [15]. In addition, we recently demonstrated that high levels of plasma FABP4, as other inflammation mediators, were associated with endothelial dysfunction assessed by peripheral artery tonometry [16,17].

Endothelial dysfunction is the first event in the pathogenesis of atherosclerosis and refers to an imbalance in the release of vasodilating molecules, such as nitric oxide (NO) and vasoconstricting factors. NO-dependent vasodilatation is thought to reflect endothelial function, and its impairment is predictive of future cardiovascular risk [18,19]. The insulin-signalling pathway in the vascular endothelium leads to the activation of endothelial nitric oxide synthase (eNOS) and an increased production of NO. This pathway involves the insulin receptor-mediated phosphorylation of insulin receptor substrate 1 (IRS1), which activates PI3-kinases that then phosphorylates and activates Akt at Ser^473^. Akt directly phosphorylates eNOS at Ser^1177^, resulting in increased eNOS activation and NO production. Under pathological conditions, proinflammatory factors cause an impairment in this particular insulin-signalling pathway in the endothelium, which promotes endothelial dysfunction [20]. This impairment might be related to the defective insulin signalling in the endothelial cells. Moreover, insulin may also activate the pro-atherogenic mitogen-activated protein kinase (MAPK) pathway in endothelial cells that leads to increases in the expression of adhesion molecules and leukocyte adhesion to the vascular endothelium. In addition, experimental animals with a vascular endothelial cell-specific insulin receptor deficiency show a reduction in the expression of adhesion molecules and eNOS mRNA [21].

Although the role of circulating FABP4 on the vascular endothelium is unknown, a recent study has revealed that the elevated expression of intracellular FABP4 in endothelial cells contributes to their dysfunction through a reduction of eNOS [22]. These data along with our own observations showing the influence of circulating FABP4 on endothelial function warrants testing the hypothesis that high levels of circulating FABP4 in altered metabolic conditions could modify the correct function of endothelial cells and cause endothelial dysfunction by impairments of the insulin-signalling pathway and NO production. If this hypothesis were correct, it would contribute to the mechanisms by which circulating FABP4 contributes to vascular endothelial dysfunction in diabetes.

## Materials and methods

### Cell culture and reagents

Human umbilical vein endothelial cells (HUVECs) were obtained from Cascade Biologics (Invitrogen Life Technologies, UK). After thawing, cells were seeded in 75-cm^2^ flasks and cultured in medium 200 according to the supplier’s recommendations. Medium 200 was supplemented with 2 % low serum growth supplement (LSGS) and 1 % Gentamicin/Amphotericin solution (Invitrogen Life Technologies, UK). The cells were placed in a humidified incubator at 37 °C and 5 % CO_2_ until there were enough cells available for experiments. The HUVEC cells were used at passage 3 in the current study.

Insulin was purchased from Sigma-Aldrich, Inc. (USA). Human recombinant FABP4 and anti-FABP4 antibody was from BioVendor (Czech Republic). The anti-eNOS, anti-phospho-eNOS (Ser^1177^), anti-Akt, anti-phospho-Akt (Ser^473^), antibodies were from Cell Signaling Technology, Inc. (Beverly MA, USA). The anti-IRS1 and anti-phospho-IRS1 (Tyr^989^) antibodies were from Santa Cruz (California, USA), and the anti-IgG-HRP antibody was from Dako (Denmark).

### Design of the studies to determine the FABP4 effects

#### Effect of FABP4 on eNOS activation

Confluent cells were starved in 1 % low serum medium (without growth factors). To determine whether FABP4 has any effect on the basal eNOS activation, we studied eNOS phosphorylation at Ser^1177^. The HUVECs were incubated with FABP4 (25–100 ng/ml) for 30 min in the endothelial starvation medium. The treated cells were rinsed with ice-cold PBS and lysed in lysis buffer, which was composed of 50 mM Tris–HCl, 150 mM NaCl, 0.1 % SDS, 1 % Nonidet, 0.5 % deoxycholate and protease inhibitors, and the cells were stored at −80 °C until they were processed. The total protein concentration was measured using a Bradford assay (BioRad, USA). Immunoblot analysis of eNOS phosphorylation at Ser^1177^ and total eNOS was performed.

We also examined the effect of FABP4 on the insulin-stimulated eNOS phosphorylation at Ser^1177^. The HUVECs were preincubated with FABP4 (25–100 ng/ml) for 30 min and were then stimulated with 600 nM insulin for 30 min in the endothelial starvation medium. We used the same protocol described above to obtain the samples. The total protein concentration was measured using a Bradford assay. Immunoblot analysis of eNOS phosphorylation at Ser^1177^ and total eNOS was also performed.

#### Effect of FABP4 on the insulin-signalling pathway

Because insulin activates eNOS through IRS1 and Akt, we investigated whether FABP4 would have any effect on the insulin-stimulated IRS1 and Akt activation. We studied the phosphorylation of IRS1 at Tyr^989^ and of Akt at Ser^473^. The HUVECs were preincubated for 30 min with FABP4 (25–100 ng/ml) before a 30-min insulin treatment (600 nM) in the starvation medium. Treated cells were collected and lysed in lysis buffer, and then the lysates were stored at −80 °C until they were processed. The total protein concentration was measured using a Bradford assay. Immunoblot analysis of IRS1 phosphorylation at Tyr^989^, total IRS1, Akt phosphorylation at Ser^473^ and total Akt was performed.

#### Immunoblot analysis of eNOS phosphorylation at Ser^1177^, total eNOS, IRS1 phosphorylation at Tyr^989^, total IRS1, Akt phosphorylation at Ser^473^, and total Akt, and FABP4

Electrophoresis and immunoblot analysis were performed using the NuPAGE Protein Analysis System (Invitrogen Life Technologies, UK). The membrane was blocked with a 2 % ECL Advance Blocking Reagent (Amersham Biosciences, Fairfield, CT) and was incubated with anti-eNOS, anti-eNOS phosphorylation at Ser^1177^, anti-Akt, anti-Akt phosphorylation at Ser^473^, anti-IRS1 phosphorylation at Tyr^989^, anti-IRS1, or anti-FABP4 antibodies. Antigen-antibody complexes were detected by incubating the membrane with an IgG-HRP antibody. The bands were visualised using ECL reagents (Amersham Pharmacia, Fairfield, CT) with the VersaDoc image system and were quantified with the Quantity One analysis software version 4.6.2 (Bio Rad, USA). The relative levels of the phosphorylated proteins were quantified after being normalised to the total proteins and were expressed as arbitrary units (AU).

#### Effect of FABP4 on eNOS mRNA expression

Upon insulin stimulation, the eNOS mRNA was increased in the vascular endothelial cells. Therefore, we examined the mRNA expression of insulin-stimulated eNOS from the FABP4-treated cell lysates. Confluent vascular cells were preincubated with FABP4 (50–100 ng/ml) for 30 min and then stimulated with 600 nM insulin for 24 hours in a supplemented medium. Total RNA was isolated from the cells using the ABI PRISM 6100 Nucleic Acid PrepStation (Applied Biosystems, CA, USA). The absorbance at 260 nm was used to measure the RNA concentration, and an absorbance ratio of 260/280 nm was used to analyse the RNA quality.

#### Real-time quantitative PCR of eNOS

Total RNA (0.5 μg) was reverse transcribed to cDNA using Random Hexamers and SuperScript II (Invitrogen Life Technologies, UK) by following the manufacturer’s protocol. TaqMan primers and probes for eNOS and GAPDH were obtained from validated and pre-designed Assays-on-Demand products (Applied Biosystems, CA, USA) and were used in real-time PCR amplifications. The mRNA expression for each gene and sample was calculated using the recommended 2^-ΔΔCt^ method. The control group (untreated cells) was defined as the calibrator in this experiment. GAPDH was used as a housekeeping gene to normalise the results of the gene of interest.

#### Nitric oxide assay

Cells were starved for 24 h with 1 % low serum medium (without growth factors) and then preincubated for 30-min with FABP4 (25–100 ng/ml) before a 30-min insulin treatment (600 nM) in the starvation medium. The supernatants were collected, and the detection of the NO_2_ and NO_3_ anions was performed with the Nitrate/Nitrite Colorimetric Assay kit (Cayman Chemical, Ann Arbor, MI) using the Griess reaction and following manufacturer’s instructions. The absorbance of the solution was read on a spectrophotometer at 540 nm. To quantify the NO production, a standard nitrate curve was generated in the same medium in which the experiments were performed. The results were expressed as fold increases with respect to the insulin treatment.

### Statistical analyses

The results are represented as the means ± SD of at least 3 separate experiments. Differences between the means were determined using a one-way analysis of variance (ANOVA), which was followed by a Dunnett’s post-hoc test for multiple comparisons. Differences were considered significant at *P* < 0.05. The GraphPad Prism 5.0 Software, Inc. was used for statistical analyses.

## Results

### FABP4 inhibits eNOS activation and NO production in HUVECs

**Figure 1:**
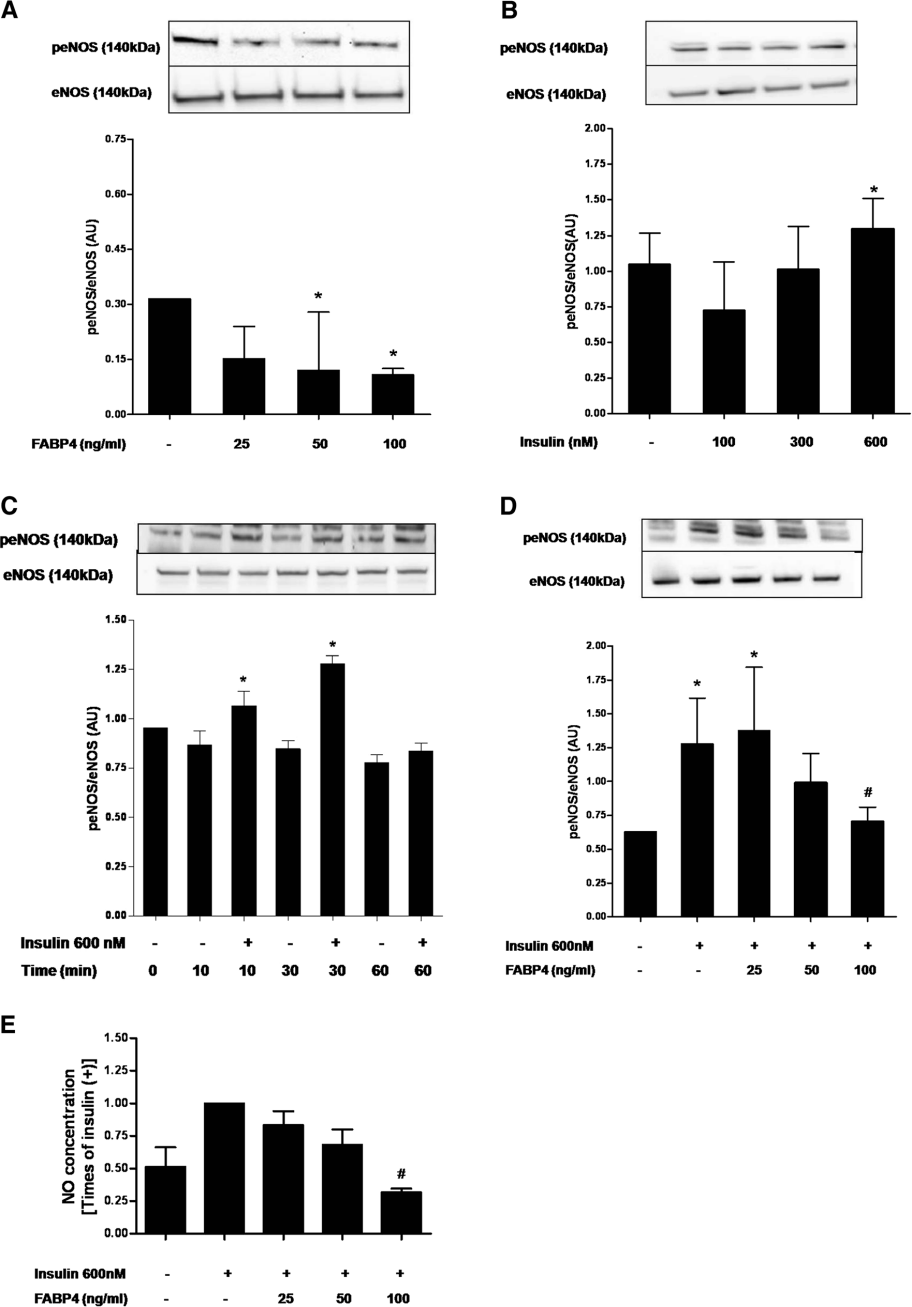
**Effect of FABP4 on eNOS activation and NO production in HUVECs. A**, effect of FABP4 on eNOS phosphorylation at Ser^1177^. HUVECs were incubated with FABP4 at the indicated concentrations for 30 minutes. **B**, eNOS Ser^1177^ phosphorylation in response to various doses of insulin (100, 300, 600 nM). HUVECs were incubated with insulin at the indicated concentrations for 30 minutes. **C**, Time course of eNOS Ser^1177^ phosphorylation in response to insulin. HUVECs were incubated with 600 nM insulin for 10, 30 and 60 min. **D**, effect on insulin-stimulated eNOS phosphorylation at Ser^1177^. HUVECs were incubated with FABP4 at the indicated concentrations for 30 minutes and then with insulin (600 nM) for 30 minutes. **E**, effect of FABP4 on NO production. HUVECs were incubated with FABP4 at indicated concentrations for 30 minutes and then stimulated with insulin (600 nM for 30 min). Representative blots are shown. The data are given as the mean ± standard deviation from three independent experiments. *P < 0.05 vs. insulin(−) / FABP4(−); ^#^*P* < 0.05 vs. insulin(+) / FABP4(−).

To determine whether FABP4 have any effect on eNOS activity, we studied the eNOS activation measuring its phosphorylation at Ser^1177^. We found that exposing HUVECs in the endothelium growth medium (with serum and growth factors that maintain the basal activation of eNOS) to FABP4 (25-100 ng/ml) inhibited eNOS phosphorylation at Ser^1177^ in a concentration-dependent manner at as early as 30-min of exposure. The maximal inhibition was achieved at 100 ng/ml of FABP4 (65 %; P < 0.05) (Figure 1A). On insulin stimulation, eNOS is phosphorylated by the activation of PI3k/Akt pathway in vascular endothelial cells. First, we performed a dose–response curve with 100, 300 and 600 nM insulin, and we observed that 600 nM insulin was required to increase eNOS Ser^1177^ phosphorylation by 20 % (*P* < 0.05) (Figure 1B). Therefore, we performed all of the experiments with 600 nM insulin. We also performed an insulin time course (10, 30, 60 min), and we observed that the maximal phosphorylation of eNOS at Ser^1177^ occurred 30 min after insulin treatment (26 % increase; *P* < 0.05) (Figure 1C). We next analysed whether FABP4 also impairs insulin–induced eNOS activation and NO production. As shown in Figure 1D, the addition of insulin increases the phosphorylation of eNOS at Ser^1177^, but a 30-min pretreatment with FABP4 (25–100 ng/ml) inhibits this insulin-dependent increase by up to 45 % with the 100-ng/ml concentration (P < 0.05) in the absence of any changes in the total protein levels. Therefore, we examined the effect of FABP4 on the ability of eNOS to produce NO under insulin-stimulated conditions. The change in eNOS phosphorylation at Ser^1177^ was accompanied by a significant decrease in the NO production of up to 68 % by treatment with 100 ng/ml of FABP4 (P < 0.05) (Figure 1E). Thus, FABP4 can inhibit both the basal and the insulin-stimulated eNOS phosphorylation at Ser^1177^ and can cause the inactivation of eNOS and decrease NO production in endothelial cells.

### FABP4 inhibits insulin-stimulated eNOS mRNA expression in HUVECs

**Figure 2:**
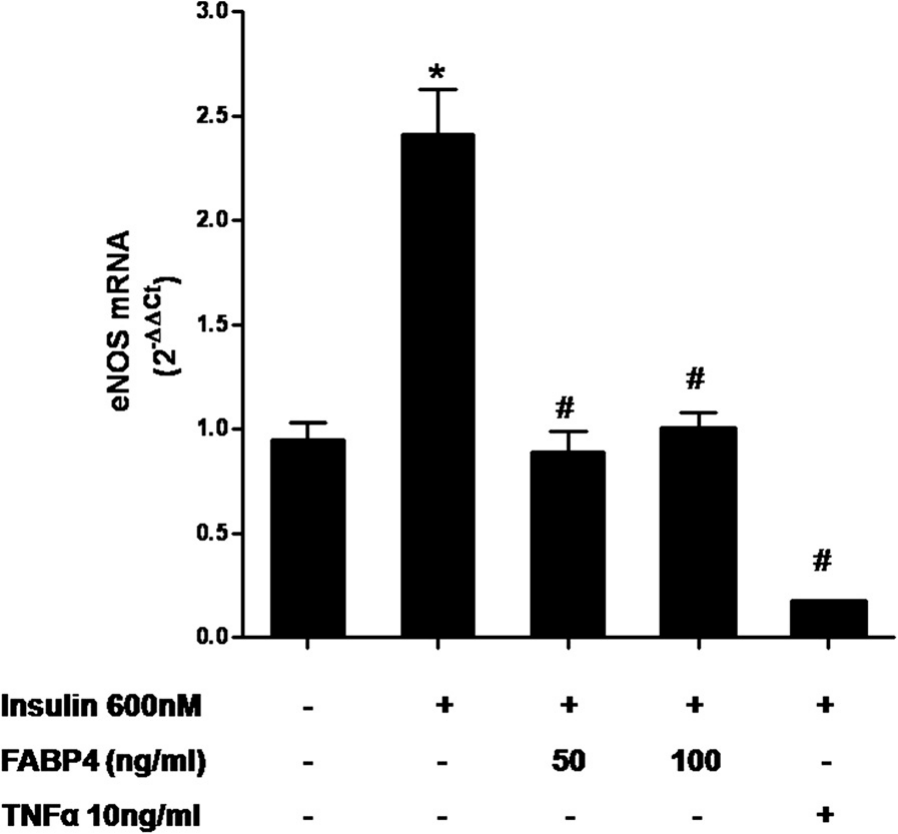
**Effect of FABP4 on eNOS mRNA expression.** HUVECs were incubated with FABP4 at the indicated concentrations for 30 minutes and then with insulin (600 nM) for 24 hours. TNFα (10 ng/ml) was used as a negative control. The data are expressed using the 2^-ΔΔCt^ method. *P < 0.05 vs. insulin(−) / FABP4(−); ^#^*P* < 0.05 vs. insulin(+) / FABP4(−).

Upon insulin stimulation, the eNOS mRNA was increased in the vascular endothelial cells. As was expected, insulin augmented the mRNA expression of eNOS after 24 hours of treatment (*P* <0.05). The upregulation of insulin-stimulated eNOS expression was inhibited by 63 % and 59 % due to the treatment with 50 ng/ml and 100 ng/ml of FABP4, respectively (*P* < 0.05) (Figure 2). Additionally, we found a 93 % decrease in the eNOS expression after an exposure to TNFα (10 ng/ml) as positive control (*P* < 0.05).

### FABP4 effect on eNOS is produced through the insulin-signalling pathway in HUVECs

**Figure 3:**
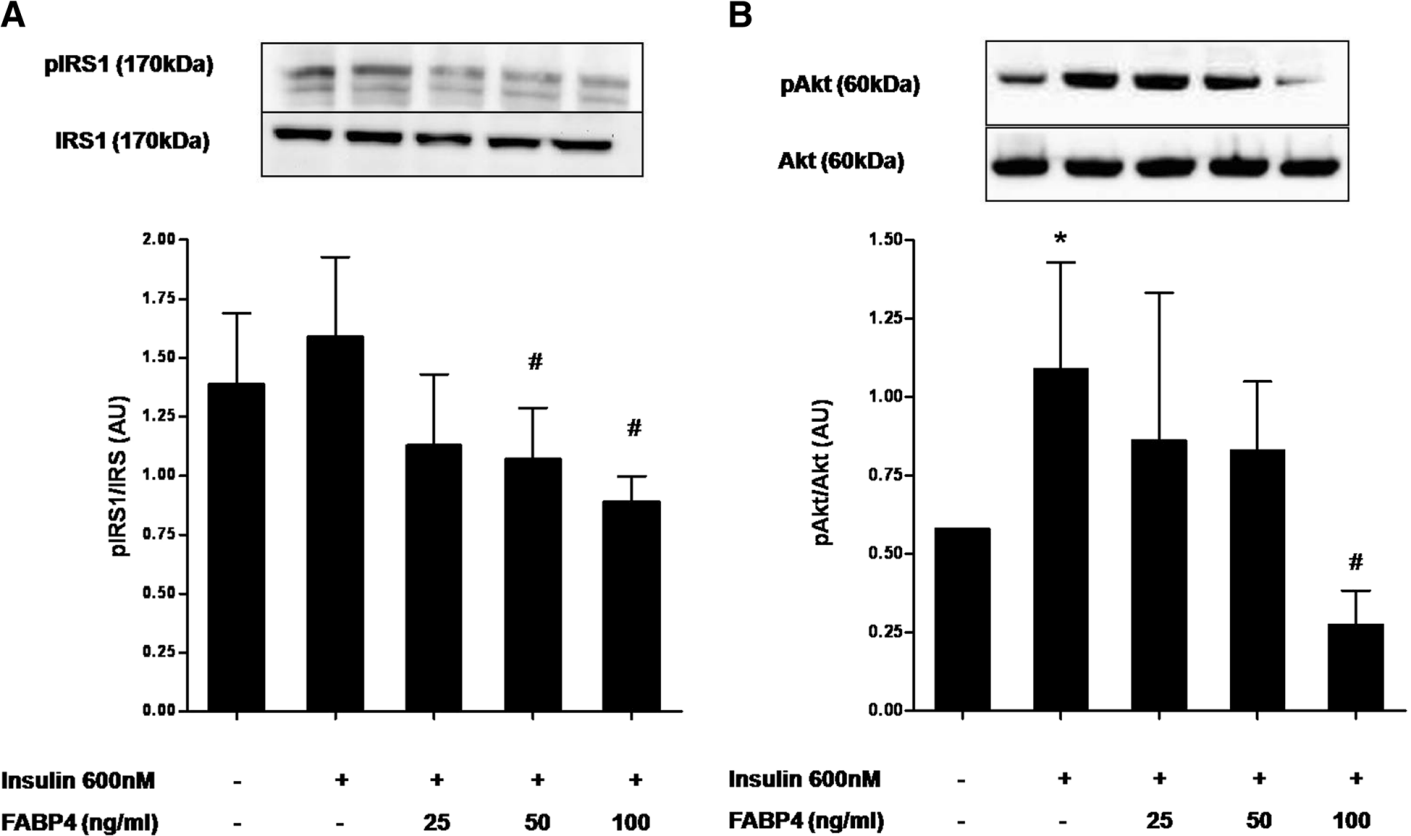
**Effect of FABP4 on IRS1 and Akt activation in HUVECs.A**, effect on insulin-stimulated IRS1 phosphorylation at Tyr^989^. **B**, effect on insulin-stimulated Akt phosphorylation at Ser^473^. HUVECs were incubated with FABP4 at the indicated concentrations for 30 minutes and then with insulin (600 nM) for 30 minutes. Representative blots are shown. The data are given as the mean ± standard deviation from three independent experiments. *P < 0.05 vs. insulin(−) / FABP4(−); ^#^*P* < 0.05 vs. insulin(+) / FABP4(−).

Since insulin activates eNOS through IRS1 and Akt pathway, we then investigated whether FABP4 have any effects on insulin-stimulated IRS1 and Akt activation, which was monitored by phosphorylation at Tyr^989^ and Ser^473^ sites, respectively. The addition of insulin to HUVECs increases about 25 % the phosphorylation of IRS1 at Tyr^989^ (Figure 3A), but a 30-min pretreatment with FABP4 (25–100 ng/ml) inhibits this insulin-dependent increase by up to 44 % with the 100-ng/ml concentration (P < 0.05). No variations were observed in the total IRS1 protein level across the different FABP4 concentration treatments. There was also a similar effect of the FABP4 treatment on the phosphorylation of Akt at Ser^473^. The insulin stimulation significantly increased the phosphorylation of Akt at Ser^473^ (Figure 3B). The FABP4 pretreatment impaired this insulin-mediated phosphorylation of Akt at Ser^473^ by up to 75 % with the 100 ng/ml concentration (P < 0.05) in the absence of any change in protein levels. Collectively, FABP4 inhibited insulin-stimulated phosphorylation of IRS1 and Akt, indicating possible impairment of upstream insulin-signalling pathway.

**Figure 4:**
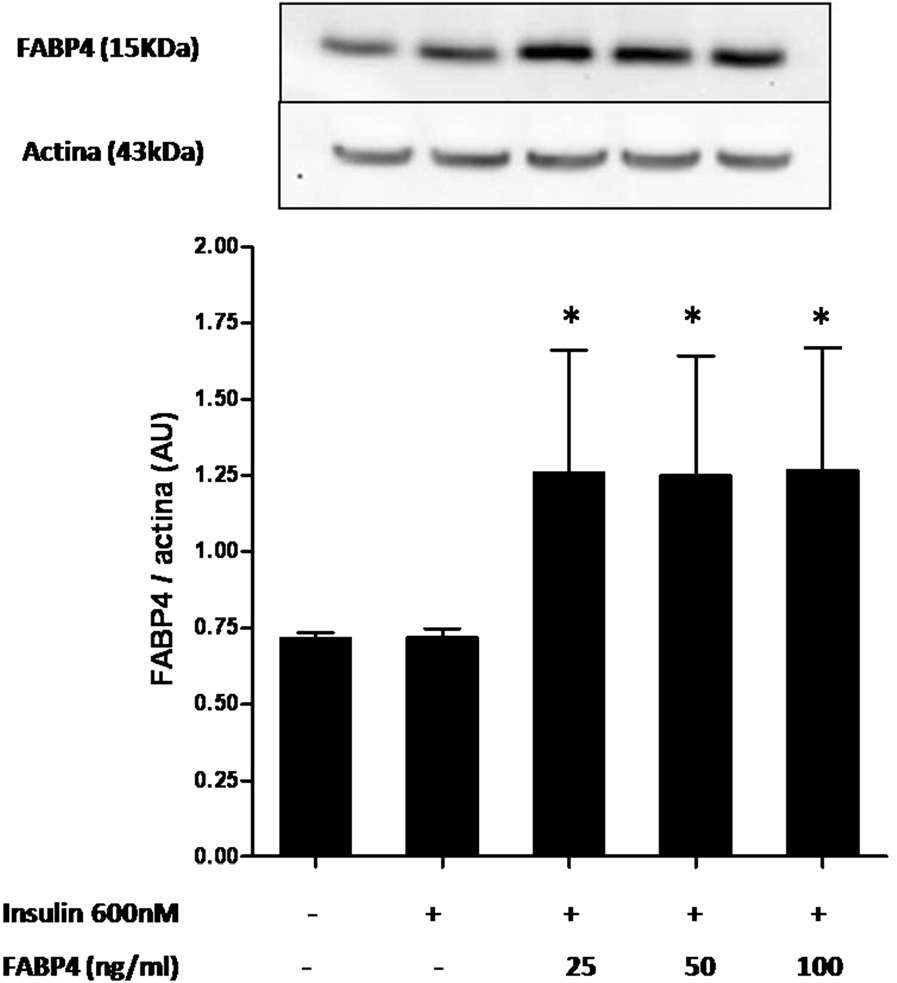
**FABP4 analysis in total cell lysates.** HUVECs were treated with the indicated concentrations of FABP4 for 30 minutes followed by 600 nM insulin for 30 minutes. Representative blots are shown. The data represent the mean ± standard deviation from three independent experiments. *P < 0.05 vs. the untreated contro.

In Figure 4, we analysed whether FABP4 was able to be internalized within the endothelial cells. We observed that incubation of HUVECs with 25–100 ng/ml FABP4 for 30 min increased the amount of exogenous FABP4 in the treated cells compared with the untreated cells (*P* < 0.05).

## Discussion

The present study demonstrates that exogenous FABP4 induces endothelial cell dysfunction *in vitro,* as assessed by the impact on one of their main properties, the vasodilatory mechanisms. We have also shown that this effect is mediated by the interaction of FABP4 with the insulin-signalling pathway in vascular cells. FABP4 alters eNOS activation, as was demonstrated by the reduction of eNOS phosphorylation at Ser^1177^ and NO production. In addition, the reduction of IRS1 phosphorylation at Tyr^989^ and Akt phosphorylation at Ser^473^ suggests that the effect observed for eNOS activation is due to the interference of FABP4 in the insulin-signalling pathway in endothelial cells. Our results support a direct effect of extracellular FABP4 on vascular cells and, therefore, a putative effect of circulating FABP4 on peripheral tissues. This observation is important because FABP4 is recognised as a biomarker of cardiometabolic risk but it could be considered as mediator of peripheral tissue damage. Several studies have linked FABP4 levels to obesity, T2D and MS [4,8,11]. Additionally, FABP4 has been associated with the burden of coronary atheromatosis, but a causal role has not yet been established. The only report that showed a direct effect of FABP4 on cells demonstrated that FABP4 reduces the contractile capacity of cardiomyocytes [15]. We have previously reported that plasma FABP4 levels are associated with endothelial dysfunction in diabetic patients [16]. Our present data supports a causal role of FABP4 in the dysfunction of the vascular wall. These findings are in accordance with a previous study suggesting that an elevated expression of intracellular FABP4 in vascular endothelial cells contributes to endothelial dysfunction both *in vivo* and *in vitro*[22]. In contrast, a recent study showed that FABP4/5 inhibitors ameliorate dyslipidaemia but not insulin resistance in diet-induced obese mice [23]. Our results show that exogenous FABP4 alters the insulin-signalling pathway at its early activation steps. Additionally, it has already been shown that FABP4 interferes with the insulin receptor [24]. Moreover, FABP4 could be phosphorylated on Tyr^19^ in response to insulin stimulation [25]. Our results support these previous observations and extend them to the exogenous FABP4, which has a greater clinical implication. Along with a decrease in eNOS expression, in our hands, FABP4 increased the expression of vascular cell adhesion protein 1 (VCAM1), E-selectin and leukocyte adhesion to endothelial cells (data not shown), suggesting a more global effect of FABP4 on endothelial function. In our experiments, HUVECs were stimulated with 600nM insulin because that was the dose with which we observed an increase in the phospho-eNOS produced and because other authors also obtained maximal nitric oxide production with similar dose [26].

The mechanisms that FABP4 utilises to interfere with the cell are currently being investigated. Although previous studies showed that Heart-FABP (62 % homology with FABP4) has the capability of binding to a membrane receptor described in cardiac cells [27], this observation has neither been confirmed nor extended to other FABP family members. It has been shown that heart FABP levels in serum could represent a useful biomarker for myocardial function in pre-diabetic patients [28]. It is not known whether FABP4 interacts directly with the insulin receptor or if it interacts with other components of the cell membrane and then secondarily modifies the insulin cascade. It is also unknown whether circulating FABP4 can be internalised into the cell to act by intracellular mechanisms. We have observed that the amount of intracellular FABP4 increases after FABP4 incubations with respect to non-treated cells, which suggests that FABP4 could be internalised by endothelial cells. FABP4 is an intracellular long-chain fatty acid transporter [1,29]. Although this function has not yet been demonstrated for circulating FABP4, we cannot exclude that the effects associated with extracellular FABP4 could be mediated by fatty acids, which are molecules known to play a role in the dysfunction of the insulin-signalling pathway. This finding could be important evidence that FABP4 plays a pivotal role in impairing the insulin signalling pathway.

Our findings suggest that high levels of FABP4 in the plasma are not just a clinical manifestation of insulin resistance but also a causative factor of the development of insulin resistance at the vascular level leading to NO metabolism alteration.

## Conclusions

In summary, we have demonstrated that FABP4 can modulate the insulin-signalling pathway in vascular cells and, consequently, decrease eNOS activation and NO production, which impairs arterial vasodilatation. Our results supports that FABP4 plays a direct causal role in endothelial dysfunction and could be a good therapeutic target for patients with T2D.

## Abbreviations

FABP4, Fatty acid-binding protein 4; T2D, Type 2 diabetes; NO, Nitric oxide; HUVEC, Human umbilical vascular endothelial cell; eNOS, Endothelial nitric oxide synthase; IRS1, Insulin receptor substrate 1; MS, Metabolic syndrome; MAPK, Mitogen-activated protein kinase; VCAM1, Vascular cell adhesion protein 1.

## Competing interests

Dr. Masana has provided lectures, consultancies and expert testimony to several pharmaceutical companies involved in lipid metabolism, such as Merk Sharp & Dohme, Roche, Esteve, Recordati and Kowa.

## Authors’ contribution

GA conducted the experimental work with contributions from JG, GA, PS, MH, AC, JG and LM contributed to method development, establishment of cell lines, experimental design and data interpretation. GA, JG and LM wrote the paper. All authors read and approved the final paper.
